# Groupwise structural sparsity for discriminative voxels identification

**DOI:** 10.3389/fnins.2023.1247315

**Published:** 2023-09-07

**Authors:** Hong Ji, Xiaowei Zhang, Badong Chen, Zejian Yuan, Nanning Zheng, Andreas Keil

**Affiliations:** ^1^The Shaanxi Key Laboratory of Clothing Intelligence, School of Computer Science, Xi'an Polytechnic University, Xi'an, China; ^2^Institute of Artificial Intelligence and Robotics, Xi'an Jiaotong Univeristy, Xi'an, China; ^3^Center for the Study of Emotion and Attention, Department of Psychology, University of Florida, Gainesville, FL, United States

**Keywords:** fMRI, groupwise regularization, voxel selection, stable hierarchical voting (SHV), randomized structural sparsity (RSS), effective vote ratio (EVR)

## Abstract

This paper investigates the selection of voxels for functional Magnetic Resonance Imaging (fMRI) brain data. We aim to identify a comprehensive set of discriminative voxels associated with human learning when exposed to a neutral visual stimulus that predicts an aversive outcome. However, due to the nature of the unconditioned stimuli (typically a noxious stimulus), it is challenging to obtain sufficient sample sizes for psychological experiments, given the tolerability of the subjects and ethical considerations. We propose a stable hierarchical voting (SHV) mechanism based on stability selection to address this challenge. This mechanism enables us to evaluate the quality of spatial random sampling and minimizes the risk of false and missed detections. We assess the performance of the proposed algorithm using simulated and publicly available datasets. The experiments demonstrate that the regularization strategy choice significantly affects the results' interpretability. When applying our algorithm to our collected fMRI dataset, it successfully identifies sparse and closely related patterns across subjects and displays stable weight maps for three experimental phases under the fear conditioning paradigm. These findings strongly support the causal role of aversive conditioning in altering visual-cortical activity.

## 1. Introduction

Machine learning approaches have become popular in cognitive neuroscience, often in the context of using neuroimaging techniques to discriminate between brain patterns associated with different experimental conditions, emotional states, cognitive processes, and ultimately health outcomes. Variable selection and feature selection have become the focus of studies using brain-based data with tens or hundreds of thousands of variables. The objective of the research addressing this problem falls broadly into two categories: (1) brain image decoding, e.g., Haxby et al. (2001) and brain-computer interface (BCI) (Wolpaw et al., 2002; Saha et al., 2021), as well as (2) multivariate hypothesis testing (Bzdok et al., 2017; Kia et al., 2017; Wen et al., 2019) including identification of candidate biomarkers for medical diagnosis (Demirci et al., 2008). The former applications pursue the maximum predictive power of the predictors, providing faster and more cost-effective predictors, while the latter put more attention on providing a better understanding of the underlying process that reflects the spatiotemporal nature of the generated data. In the present study, we are interested in the second application, i.e., brain decoding. We specifically address the problem of identifying the brain activity patterns that are associated with specific behavior. The classic univariate analysis typically models each response channel separately, which is inconsistent with the multivariate nature of neuronal population codes and also with the observation that noise is spatially correlated. Separate modeling of each response entails low power for testing and comparing models, for two reasons: (1) Single fMRI responses may be noisy, and the evidence is not combined across locations. (2) The analyses treat the responses as independent, thus forgoing the benefit exploited by linear decoding approaches to model the noise in a multivariate manner. This is particularly important in fMRI data analysis, where nearby voxels have highly correlated noise. As spatial resolution increases, we face the combined challenge of increasing the number of individual voxels (inflating the feature space) and also increasing the noise in those individual voxels.

In order to understand the learning process of human in response to an initial neutral visual stimulus predicting an aversive outcome, we conducted a study using fMRI to observe the large-scale neurophysiological changes. In neuroimaging, a decoder is a predictive model that, given a series of brain images, fits the binary classification information regarding an experimental condition, a stimulus category, a motor behavior, or a clinical state. In the context of aversive conditioning, one of two initially harmless stimuli [referred to as conditioned stimuli (CS)] acquires motivational significance by consistently predicting the occurrence (CS+) of a negative event [known as the unconditioned stimulus (US)], while the other stimulus (CS−) predicts its absence. Since US is generally a noxious stimulus, it is difficult to obtain satisfactory sample sizes for such psychological experiments, given the tolerability of the subjects already ethical considerations. Therefore, we here focus on linear brain decoding because of its broader usage in analyzing inherently small sample size (Pereira et al., 2009). The estimated classification or regression weights can be visualized in the form of brain maps, which can aid in understanding how brain activity in space and time underlies a cognitive function (Mourao-Miranda et al., 2005). Selecting an appropriate set of voxels as the input for the classifier construction is of critical importance. The voxels corresponding to the non-zero weights are considered as the relevant features. The identification of discriminative voxels is based on the values of the weight vector, and their importance is proportional to the absolute values of the weights.

Due to the high-dimensionality of neuroimaging, high correlations among different voxels and low signal-to-noise ratios (SNRs), multiple weight maps yielding the same predictive power. In other words, different models lead to very similar generalization performance, and the recovered brain maps often suffer from lack of interpretability. Therefore, improving the interpretability of brain decoding approaches is of primary interest in many neuroimaging studies, especially in a group analysis of multi-subject data. At present, there are two main approaches proposed to enhance the interpretability of multivariate brain maps, as reviewed by Kia et al. (2017): (1) Introducing new metrics into the model selection procedure. (2) Introducing new hybrid penalty terms for regularization. The first approach to improving the interpretability looks for the best values for the hyper-parameters of a model (Lemm et al., 2011; Hoyos-Idrobo et al., 2018). The second approach involves applying regularization or prior knowledge (Zou and Hastie, 2005; Yuan and Lin, 2006; Rasmussen et al., 2012) to restrict model complexity, also known as dimension reduction. This approach is commonly used for the ill-posed nature of brain decoding problems (Geman et al., 1992).

As a representative of the second category, structured sparsity models (Chambolle, 2004; Bach et al., 2012; Micchelli et al., 2013) extend the least absolute shrinkage and selection operator (LASSO) model by promoting sparse models in some preferred way. For example, regression weights may be encouraged to be constant or vary smoothly within regions of the brain (Michel et al., 2011; Baldassarre et al., 2012; Gramfort et al., 2013). Despite the fact that sparsity has traditionally been connected with interpretability, these structured sparsity models incorporating additional spatial constraints into the predictive model, allowing for even greater ease of interpretation by further grouping the discriminative voxels into few clusters based on prior information (Yuan et al., 2011; Li et al., 2014; Shimizu et al., 2015). Besides, stability selection is applied as an effective way to control the false positives (Meinshausen and Bühlmann, 2010; Ye et al., 2012; Shah and Samworth, 2013; Cao et al., 2014; Rondina et al., 2014; Wang and Zheng, 2014). While the control of false positives can be achieved, a significant false negative rate is often expected, especially in the case of redundant and correlated voxels, this correlation prior is not explicitly taken into consideration. In Wang and Zheng (2014) and Wang et al. (2015) the authors proposed a “randomized structural sparsity”, incorporating the idea of structural sparsity in the stability selection framework, together with the subsampling scheme which further help to refine and outline the exact shapes of the discriminative regions. These regions may not be the same size as the prior partitions, which is crucial for neighboring voxels belonging to the same brain area. Although they may be highly correlated, not all neighboring voxels are necessarily significant discriminative voxels (Witten et al., 2014). A similar strategy was used in Wan et al. (2014) and Yan et al. (2015) to predict cognitive outcomes via cortical surface measures. The results showed improved decoding accuracy and interpretability of brain maps.

In order to enhance the stability and reproducibility of our model during optimization, we apply group constraints and regularization across multiple subjects. This technique is commonly used in transfer learning or multitask learning (Bakker and Heskes, 2003; Raina et al., 2006; Dai et al., 2007; Pan and Yang, 2010). In our paper, we make the assumption that the regions of discriminative voxels are relevant or overlapping to a certain extent across subjects. Additionally, we assume that only a few clusters are actually discriminative for the classification problem. To achieve these goals, we propose to use a mixed *l*1 and groupwise *l*2 norm for regularization. The *l*2 norm penalizes large coefficients and yields a non-sparse weight distribution inside the group, while the *l*1 norm promotes sparsity on selected clusters. This nested mixed-norm regularization enables us to construct stable and interpretable models by pooling data from multiple subjects. It is important to note that the *l*2 norm does not imply the application of unified weights to the functionally significant clusters, which might be a too strong constraint and impractical for the real data.

Based on stability selection and the groupwise structural, we propose a stable hierarchical voting (SHV) mechanism to monitor the quality of spatial random sampling and reduce the risk of false and missed detections. When using uniform sampling, there is a possibility that many noisy and uninformative voxels will be included. To address this issue, we use multiple cross-validations of test accuracy during the voting process to select high-quality samples. In addition, small perturbations in the observations can cause instability in the model generated (Arlot et al., 2010). To mitigate this problem, we apply model averaging to aggregate the output of multiple models as suggested (Nemirovski, 2000). Furthermore, the number of selected candidate features is allowed to be much larger when incorporating group structure (Jenatton et al., 2011; Xiang et al., 2015), which allows us a more global search among brain regions.

## 2. Methods

### 2.1. Pre-segmentation

For the class of methods that use structural information for dimensionality reduction, the number of clusters to be generated is estimated based on finding a compromise between several factors: (1) To enhance area homogeneity, it tends to conduct fine segmentation for small patches. (2) To avoid the false negative selection due to spatial sparsity induced by the *l*1 norm, it tends to perform rough segmentation for large patches. (3) The number of trials is taken into consideration as the unknowns of the optimization problem is now equal to the number of clusters. From the previous study (Craddock et al., 2013), with 200 ROIs, the resulting parcellations consist of clusters with anatomic homology and thus offer increased interpretability.

In our work, we first obtain the structural information about the brain according to their strong local correlations. Here we perform a data-driven segmentation operation to partition the voxels into small clusters using the normalized cut (NCut) (Shi and Malik, 2000; Cour et al., 2005). To define the affinity between two voxels *v*_1_ and *v*_2_ we combine three cues: (1) the correlations of the raw BOLD time series, (2) the correlations of BOLD features for each trial, (3) a connection radius σ_*d*_ to attenuate the influence from far away voxels. Voxels in close proximity with similar BOLD waveforms are likely to be part of the same cluster. Additionally, incorporating correlations among features helps to minimize the impact of signal clutter. Furthermore, averaging the features results in a fit with lower variance compared to individual features, especially when they are positively correlated (Park et al., 2006; Wang et al., 2015). This aspect also contributes to the potential enhancement of stronger features.

The affinity matrix is computed based on finding the combined data from multiple subjects since uniform segmentation is required for group-wise regularization. Let us denote the preprocessed fMRI data matrix as $\tilde{X}\in {\mathbb{R}}^{N_{t}\times N_{V}}$, where *N*_*t*_ is the number of scans, *N*_*V*_ is the number of voxels. To access the columns of a matrix, the *v*-th column is denoted as (:, *v*). We construct the affinity matrix ***A*** as follows:


$$\begin{array}{l} A_{v_{1},v_{2}}=|corr\left(\tilde{X}\left(:,v_{1}\right),\;\tilde{X}\left(:,v_{2}\right)\right)|\cdot \exp\left(-dist{\left(v_{1},v_{2}\right)}^{2}/\sigma_{d}^{2}\right) \end{array}$$

where |·| gets the absolute value, *corr*(·, ·) is the correlation between two variables, and *dist*(·) evaluates the Euclidean distance of two voxels in 3D space.

### 2.2. Classification using groupwise structural sparsity

Let us denote the feature matrix from subject *i* as $X^{i}\in {\mathbb{R}}^{N_{T}\times N_{V}}$, *i*∈{1...*N*_*S*_}, where *N*_*T*_ is the number of trials, *N*_*V*_ is the number of voxels, and *N*_*S*_ is the number of subjects. For this study, we are interested in classifying the experimental conditions. We denote the binary labeling information as $y\in {\mathbb{R}}^{N_{T}}$, ***y***(*t*)∈{1, −1} that correspond to the CS+ and CS− conditioning, respectively. The stability sampling is performed in terms of the subsampling on the features, i.e., the columns of ***X*^*i*^**, as well as subsampling of the observations, i.e., the rows of ***X*^*i*^**. Then parceling information is used to average the features within a cluster. We denote the set of the clusters via the pre-segmentation as G, and denote the number of clusters as *N*_*C*_. Specifically, each cluster $g_{j}\in G$, consists of highly correlated neighboring voxels, the sampled voxels lying in cluster *j* are noted as a set ${g_{j}}^{\prime }\subset g_{j}\in G$, for each chosen trial *t*, and ***D***(*t, j*) is the corresponding average of $X\left(t,g_{j}^{{\;}^{\prime }}\right)$ of cluster *j*. The model can be simplified to the following low dimensional problem.


(1) $$\begin{array}{c} F=\arg\underset{w}{\min}\sum_{t=1}^{N_{T}}log\left(1+exp\left(-y(t)\left(D(t,:)w+b\right)\right)\right)\\ +\lambda \sum_{j=1}^{N_{C}}∥w(j)∥ \end{array}$$

where $w\in {\mathbb{R}}^{N_{C}}$ is the weight vector. **w**(*j*) denotes the weight of *j*-th cluster, corresponding to the subset $g_{j}\in G$. The voxels corresponding to weight with large absolute value are considered as discriminative voxels (Wang et al., 2015).

In this paper, we propose to consider a group of subjects together and constrain the model using a mixed *l*_1_/*l*_2_ norm. We combine the weight vectors from all subjects into a matrix $W\in {\mathbb{R}}^{N_{C}\times N_{S}}$. Correspondingly, the objective of the model is below:


(2) $$\begin{array}{l} F=\arg\underset{W}{\min}\overset{N_{S}}{\underset{i=1}{\sum }}\overset{N_{T}}{\underset{t=1}{\sum }}log\left(1+exp\left(-y^{i}\left(t\right)\left(D^{i}\left(t,:\right)W\left(:,i\right)+b^{i}\right)\right)\right)\\ +\lambda \overset{N_{C}}{\underset{j=1}{\sum }}∥W\left(j,:\right)∥ \end{array}$$

**Figure 1:**
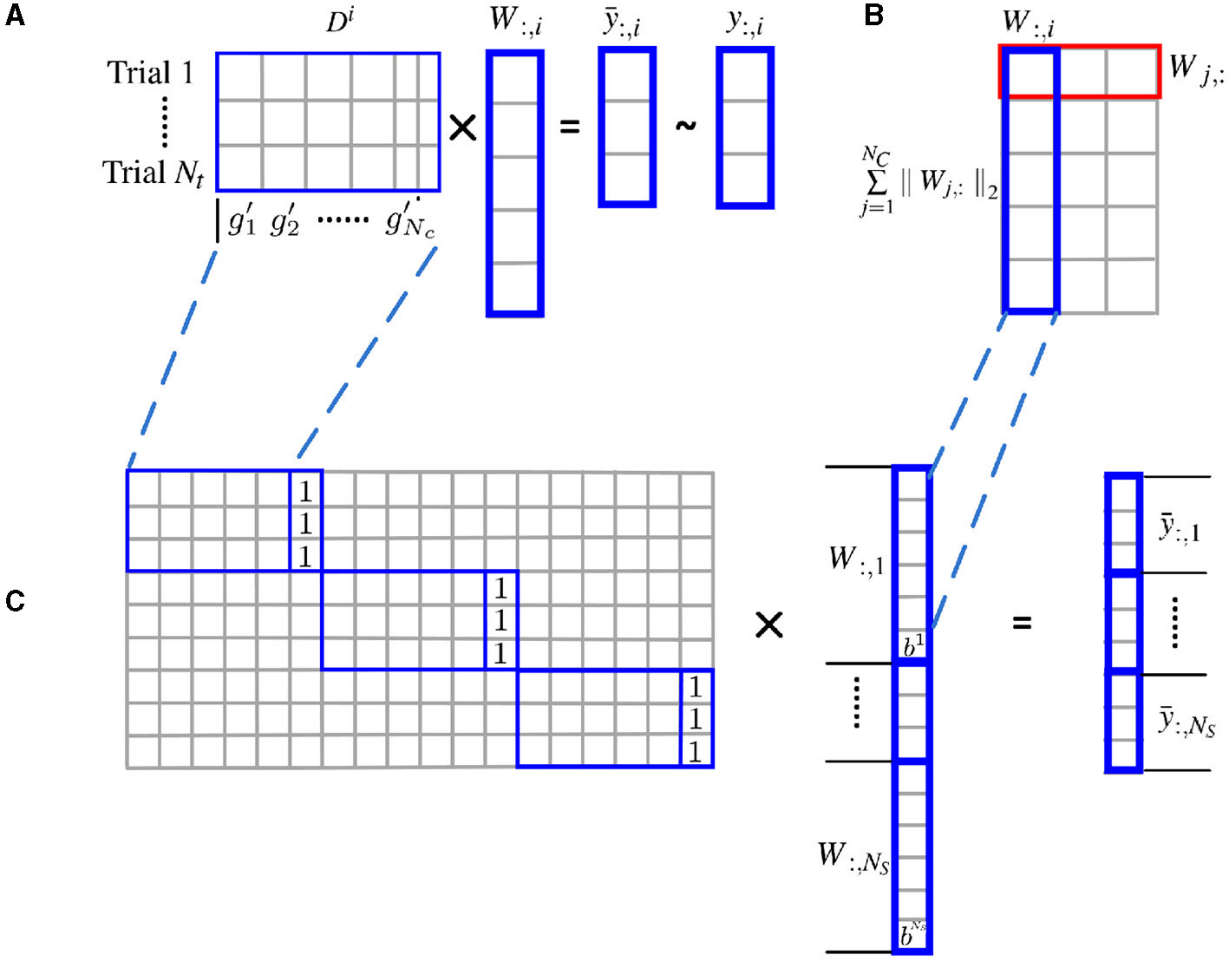
Estimate cluster weights using joint optimization of multiple subjects with *l*2 norm group constraint. **(A)** Structural sparsity model of single subject; **(B)** groupwise structural constraint using mixed *l*_1_/*l*_2_ norm; **(C)** for the convenience of optimization, the weight matrix ***W*** is vectorized, and the individual feature matrix ***D***^*i*^ are incorporated to form a block diagonal matrix with an additional column of all 1. The label information is merged from all subjects accordingly.

As shown in Figure 1, the *l*2 norm over multiple subjects for each cluster is proposed as a group constraint, i.e., the rows of ***W*** shown in the red box of Figure 1B, while the *l*1 norm on clusters further enforces structural sparsity on the solution. Using the mixed *l*1 and *l*2 norm as a joint optimization criterion allows the pooling of data from multiple subjects and enforces consistency of the selection of clusters across subjects. For the convenience of optimization, the weight matrix is vectorized, and the individual feature matrix and the label information are integrated from all subjects accordingly.

Note that the number of clusters obtained is typically much smaller than the number of voxels (*N*_*V*_) and comparable to the total number of total samples. By reducing the number of unknowns and integrating data from multiple subjects, we are able to use fewer samples to estimate the parameters.

### 2.3. Algorithmic framework

Unlike the general stability selection framework (Meinshausen and Bühlmann, 2010; Shah and Samworth, 2013; Wang et al., 2015), our algorithm, stable hierarchical voting (SHV), represents a step further with stricter control for model variance among subjects. The detailed description is outlined in Algorithm 1. Based on stability selection and the groupwise structural constraint, SHV employs a stable hierarchical voting mechanism to monitor the sample quality of spatial random sampling and reduce the risk of false and missed detections. The proposed method utilizes a two-level nested loop approach to construct a predictive decoding model for multi-subject data, while considering mixed regularity constraints. The outer loop randomly samples voxels and performs dimensionality reduction feature expressions on the corresponding motifs; The inner loop assesses the predictive ability of these features, by computing the average prediction correctness through cross-validation. Subsequently, the outer loop performs cumulative voting on the selected voxel samples, based on their prediction ability as evaluated by the inner loop. This structuring guarantees that only votes with high test precision are considered.

**Algorithm 1:** The algorithm framework of groupwise structural sparsity for discriminative voxel identification.

**Require:** Dataset of subject *i*: $X^{i}\in {\mathbb{R}}^{N_{T}\times N_{V}}$, *i*∈{1..*N*_*S*_}; Label information $y\in {\mathbb{R}}^{N_{T}}$, where *N*_*T*_ is the number of trials, *N*_*V*_ is the number of voxels; Predefined parcellation G; Groupwise sparsity penalization parameter λ; Loops of spatial randomizations *N*_*K*_; Loops of cross verification *N*_*L*_; Subsampling ratio α_*row*_, α_*col*_ in terms of rows and columns of ***X***; Minimum acceptable precision *p*; Sampling quaility control ratio β; The number of clusters one wish to select *N*_*sel*_;
**Ensure:** Effective vote ratio (EVR) for each voxel.
1: **for** *k* = 1 to *N*_*K*_ **do**
2: **for** *l* = 1 to *N*_*L*_ **do**
3: **for** *i* = 1 to *N*_*S*_ **do**
4: Perform subsampling on voxels (columns of ***X*^*i*^**) and calculate the averaged feature matrix: $D^{i}\leftarrow X_{\left[:,I\right]}^{i}\leftarrow X^{i}$, where I⊂ {1, 2, ⋯ , *N*_*V*_}, $D^{i}\in {\mathbb{R}}^{N_{T}\times N_{C}}$.
5: Perform subsampling on trials (rows of ***X*^*i*^**): $D_{\left[J,:\right]}^{i}\leftarrow D^{i}$ and update $y_{\left[J\right]}\leftarrow y$, J⊂ {1, 2, ⋯ , *N*_*T*_}.
6: **end for**
7: Estimate ***W*** with Equation 2.
8: **end for**
9: **for** *i* = 1 to *N*_*S*_ **do**
10: Calculate the average test accuracy $R_{i,k}^{test}$ across all the cross-verification loops.
11: **end for**
12: **end for**
13: **for** *i* = 1 to *N*_*S*_ **do**
14: Select *N*_*i*_ well sampled loops out of *N*_*K*_ loops according to $R_{i,k}^{test}$
15: **for** *k* = 1 to *N*_*i*_ **do**
16: Compute the score vector ***s***_*i, k*_ with Equation 3.
17: Select the *N*_*sel*_ clusters with highest coefficients in ***s***_*i, k*_.
18: **end for**
19: **end for**
20: Compute the effective vote ratio $\phi_{i}^{V}$ according to Equation 6.

In the following, *i* denotes the subject index, *i* = {1, 2, ⋯ , *N*_*S*_}, *j* denotes the cluster index, *j* = {1, 2, ⋯ , *N*_*C*_}, and *m* denotes the voxel index, *m* = {1, 2, ⋯ , *N*_*V*_}. For the outside layer, we perform constrained block subsampling in terms of voxels (columns) and calculate the averaged feature matrix, the number of resamplings denotes as *N*_*K*_. Let the subsampling fraction be α_*col*_∈(0, 1) and I denotes the set of voxel indices randomly picked.

To avoid instabilities of the generated model caused by perturbations of the observed data, we apply model averaging to mitigate this problem (Nemirovski, 2000; Arlot et al., 2010). For loop *k*, the weight vector for *l*th cross-verification is denoted as ***W***_*l*_(:, *i*), the score vector ***s***_*i, k*_ is calculated by the following equation


(3) $$\begin{array}{l} s_{i,k}\left(j\right)=\frac{1}{N_{L}}\overset{N_{L}}{\underset{l=1}{\sum }}|W_{l}\left(j,i\right)|,\;j=\left\{1,2,\cdots \;,N_{C}\right\} \end{array}$$

where |·| get the absolute value, and *N*_*L*_ denotes the number of cross-verification, which is usually chosen according to the sample size and balance with the computation cost.

We hierarchically define the selectors, from cluster to voxel, respectively. Let π(*, *N*_*sel*_) be the operation to select the top *N*_*sel*_ non-zero coefficients from vector *, and return the selector by marking the selected components to be unit valued (zero valued for the non-selected ones). If the actual non-zero components is less than *N*_*sel*_, less components are selected.

Because uniform sampling is likely to include many noisy and uninformative voxels, for *N*_*K*_ times of spatial resampling, we only count *N*_*i*_ loops when the test accuracy of cross verification go above the sampling quality control factor *q*. The number of selected loops is determined based on a quality control ratio α_*K*_∈(0, 1), only the top [α_*K*_*N*_*K*_] loops with the highest test accuracy are taken into consideration.

For group-level statistical inference, we compute the cluster-wise voting rates **ϕ**^*C*^ that incorporate the votes from multiple subjects


(4) $$\begin{array}{l} \phi^{C}=\frac{1}{N_{S}}\overset{N_{S}}{\underset{i=1}{\sum }}\pi \left(\frac{1}{N_{i}}\overset{N_{i}}{\underset{k=1}{\sum }}\pi \left(s_{i,k},N_{sel}\right),N_{sel}\right) \end{array}$$

We accumulate the votes of all the qualified selectors and then normalize the value with the sampling times of the voxel. Given that a sampled voxel *m* that belongs to cluster *j*, the voting rate of $\phi_{i}^{V}$ is defined as


(5) $$\begin{array}{l} {\tilde{\phi }}_{i}^{V}\left(m\right)=\frac{\sum_{k=1}^{N_{i}}\delta \left(m\in I_{k}\;\&\;\pi \left(s_{i,k},N_{sel}\right)\left(j\right)==1\right)}{\overset{N_{i}}{\underset{k=1}{\sum }}\delta \left(m\in I_{k}\right)},\;m\in g_{j} \end{array}$$

To ensure the stability and reliability of voting, the effective vote ratio (EVR) is defined as


(6) $$\begin{array}{l} \phi_{i}^{V}\left(m\right)={\tilde{\phi }}_{i}^{V}\left(m\right)\cdot \phi^{C}\left(j\right),\;m\in g_{j} \end{array}$$

We chose the regularization parameter λ in Equation (2) that maximize the averaged prediction accuracy below.


(7) $$\begin{array}{l} \bar{R}=\frac{1}{N_{S}}\overset{N_{S}}{\underset{i=1}{\sum }}\left(\frac{1}{N_{i}}\overset{N_{i}}{\underset{k=1}{\sum }}R_{i,k}^{test}\right) \end{array}$$

### 2.4. Stability evaluation

We adopt the stability index defined by work Baldassarre et al. (2017) to evaluate the stability of our results on real fMRI across multiple subjects. The voxels selected by EVR for subject *i* are denoted as $S_{i}=\left\{m|\phi_{i}^{V}\left(m\right)\neq 0\right\}$. Consider two sets of selected voxels, namely *S*_1_ and *S*_2_. The corrected pairwise relative overlap is calculated using the formula:


(8) $$\begin{array}{l} O\left(S_{1},S_{2}\right)=\frac{\left||S_{1}\cap S_{2}|-|S_{1}|*|S_{2}|/N_{V}\right|}{\max\left(\left|S_{1}|,|S_{2}\right|\right)} \end{array}$$

Here, |*S*_1_∩*S*_2_| is the number of voxels that are present in both sets, while |*S*_1_|*|*S*_2_|/*N*_*V*_ represents the expected number of overlapping voxels between two random samples of size |*S*_1_| and |*S*_2_| respectively, where *N*_*V*_ is the total number of voxels. The average pairwise overlap $\bar{O}$ is obtained by taking the average of the relative overlap values of all pairs of subjects.

## 3. Results

### 3.1. Synthetic data

To test and analyze the proposed algorithm on a similar problem scale as the real fMRI data, we work on a 53 × 63 × 52 brain image that has 173,628 voxels of interest. Specifically for small-sample fMRI data, we assume only 40 training 20 CS+ trials and 20 CS− trials since fMRI datasets of this size are most commonly found in psychological paradigm validation sessions. For the simulations, we use the Automated Anatomical Labeling (AAL) atlas template that segments the brain into 116 anatomical regions (Tzourio-Mazoyer et al., 2002), commonly used for different types of functional and anatomical analysis of neuroimaging data. To test whether our algorithm has superior discriminative power, we assume that there is a linear combination of a portion of voxels with categorization ability in three brain regions that have some overlap in different individuals. Specifically, all subjects were assumed to have a functional network of three distributed discriminative brain regions *G*_1_ = {32, 44, 62}, comprising three brain regions in the frontal, parietal and occipital lobes, each including over 300 discriminative voxels. Considering the complexity of the brain functional network and dramatic individual differences among subjects, we define 15 interference regions for each individual, and the interfering brain regions were not exactly the same for different individuals. For subject *i*, we define individual interference region set $G_{0}^{i}=\left\{t\;|\;\left(72+i\times 3\right)\leq t\leq \left(86+i\times 3\right)\right\}$, which are all continuous sets with 15 and three regions skipped between two sets. Each region contains roughly 300 voxels.

The base value of elements $M_{j}^{i}$ in both discriminative regions and interference regions are generated from the standard uniform distribution *U*(0, 1), where *j* = 1, 2, …, 116 representing the index of regions, other voxels in the brain image are noise generated by a standard Gaussian distribution. For discriminative regions *G*_1_ we simulate a spatially distributed pattern constrained by linear model $y_{1}^{i}=\underset{j\in G_{1}}{\sum }{\tilde{W}}_{j}^{i}\cdot M_{j}^{i}$, and samples of CS+ fall in the top 40% and CS- fall in the bottom 40% of the overall distribution of *y*_1_, therefore the simulated data can be distinguished by the linear classifier. The weight ${\tilde{W}}_{j}^{i}$ is scaled by a personalized factor $\alpha_{j}^{i}$ that allows different connectivity strength ${\tilde{W}}_{j}^{i}=W_{j}^{init}\cdot \alpha_{j}^{i}$, where $W_{G_{1}}^{init}=\left\{1,1,-2\right\}$ and $\alpha_{j}^{i}\sim U\left(0.5,1.5\right)$ that uniformly distributed with minimum 0.5 and maximum 1.5. For interference regions *G*_0_ we simulate $y_{0}^{i}=M_{j}^{i}$ and samples of CS+ fall in the top 80% and CS− fall in the bottom 80% of the overall distribution. At last, gaussian noise is added to generate observations for single trials and single voxels $x_{t,v}^{i}=y_{j}^{i}+\epsilon_{t,v},\epsilon_{t,v}\sim N\left(0,1\right)$, where *t* denotes the index of trials and *v* the index of voxels.

The elements in discriminative and interference regions are both random samples from the uniform distribution; therefore, a single region should have no significant correlation with labels in absence of noise. On the contrary, the linear combination of regions in *G*_1_ is discriminative, whereas for *G*_0_, it is not. It is noticeable that although the discriminative areas are common for all subjects, the coefficients vary for each subject. Intentionally, we added noise to simulate the case that the interference regions may have an equal or even stronger degree of correlation by chance, which would result in false positives. Such simulation is crucial, especially for studies with few samples. In the following, we conducted several experiments on the synthetic data to examine the performance of the proposed algorithm.

### 3.2. Ablation study

For the ablation study, we compare experimental results with and without applying the proposed multi-subject *l*2 norm group constraint and test the effect of the algorithm on the choice of hyper parameters, including the effect of choosing different λ and *N*_*sel*_ on the results for selected discriminative clusters. In the following, we use the following notation:

- Our proposed method: estimate cluster weight using joint optimization of multiple subjects with the proposed Algorithm 1 and Equation (2);
- Alternative method: perform the same procedure of constraint block sampling and in terms of voxels and sub-sampling cross verification in terms of trials, then estimate cluster weight for each subject separately using Equation (1);

For the block bootstrap sampling methods, block size might affect the performance of the algorithm (Lahiri et al., 1999). Given the number of blocks, there are inherent trade-offs in the choice of block size. When only minimal loops of randomizations are allowed, the choice of large blocks is likely not matching the indeed supported geometry and are prone to many false positives, while the choice of small blocks may result in many false negatives due to ignorance of local correlation of adjacent voxels (Wang et al., 2015). Empirically we chose 3 × 3 × 3.

We accumulate one vote for the identified discriminative clusters corresponding to the top four weights with largest magnitude, then summing up all the votes across subjects. Although regularization helps to reduce model variance and larger regularization parameter (λ in Equations 1, 2) yields models with more degree of sparsity and fewer sets of selected variables (clusters), we tested how λ influence the outcome of selected discriminative clusters in both alternative method and our proposed method. Please note that the proposed method and the comparison method have different objective functions. Therefore, we employ two sets of lambda values, each consisting of one larger lambda and one smaller lambda. This is intended to showcase the influence of Lambda tuning on the outcomes.

**Figure 2:**
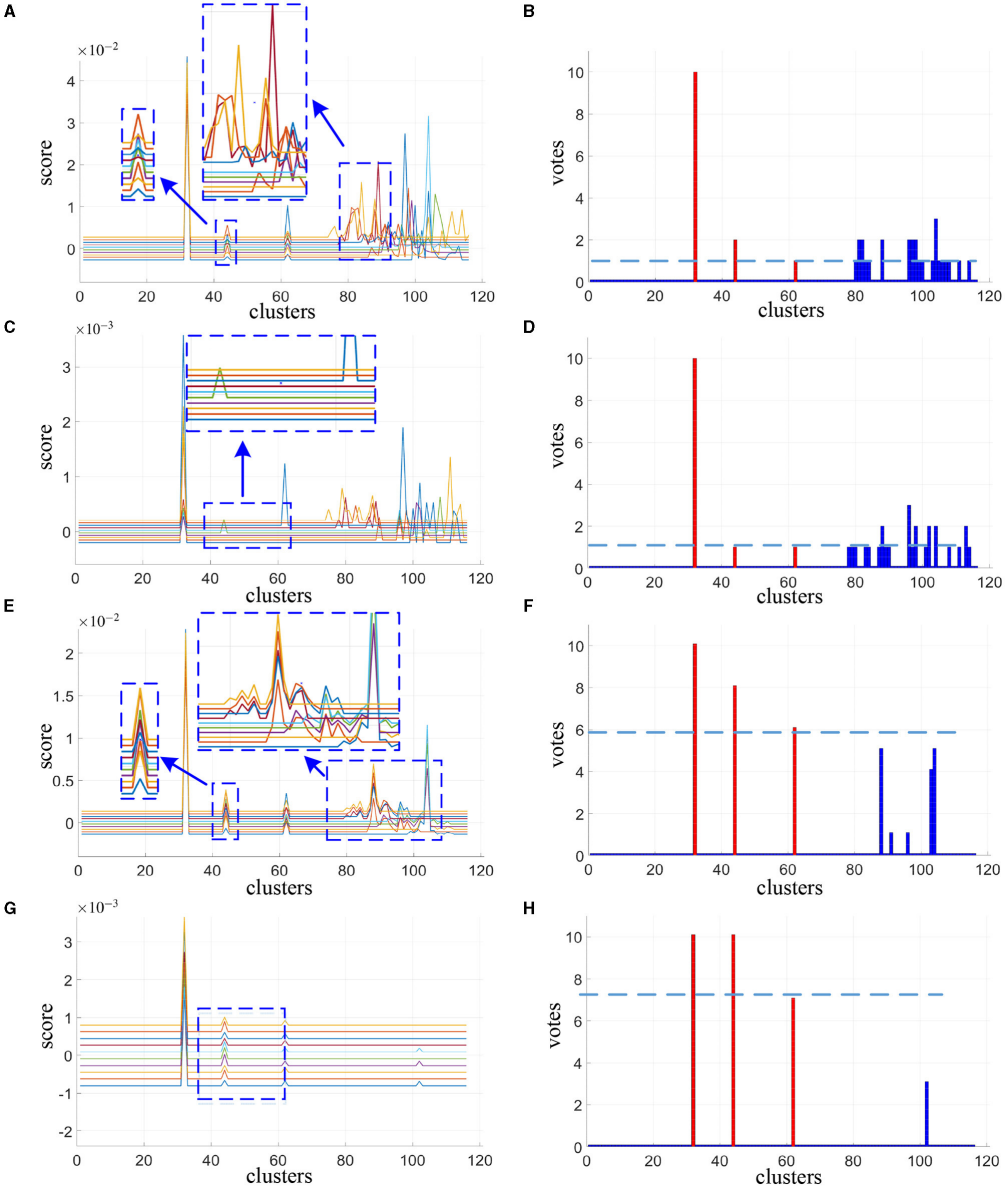
Cluster scores and vote results as estimated by alternative method at λ = 30 **(A, B)** and λ = 120 **(C, D)** and our proposed method at λ = 10 **(E, F)** and λ = 30 **(G, H)**. For cluster scores **(A, C, E, G)**, the blue arrows indicate the enlarged view of the original image, each colored line represents the result of one subject. For vote results **(B, D, F, H)**, the red lines indicate the true discriminative clusters, and the blue lines indicate the interference clusters.

The cluster scores reported in Figures 2A, C, E, G are averaged from 200 spatial subsampling steps each of which has 20 times cross validation, and the corresponding voting results are reported in Figures 2B, D, F, H. In Figure 2A we can see that for the alternative method, numerous interference clusters get higher scores than the true discriminative clusters. Larger λ, as shown in Figure 2C, helps to reduce false positives, however also increases false negatives. For the corresponding votes there is no single thresholding to distinguish discriminative clusters from the interference clusters, as can be seen in Figures 2B, D. For our proposed method, in Figure 2E as we can see from the enlarged view, scores estimated for discriminative cluster 44 are more consistent across subjects compare to the alternative method in Figure 2A, and the scores for interference clusters are relatively more sparse. As the λ increases, the score of the interference regions attenuated more significantly than the discriminative regions, as depicted in Figure 2G. Meanwhile, as shown in Figures 2F, H, there exist proper thresholds to separate all the three discriminative clusters correctly, and sparsity helps to increase the classification gap between the two.

**Figure 3:**
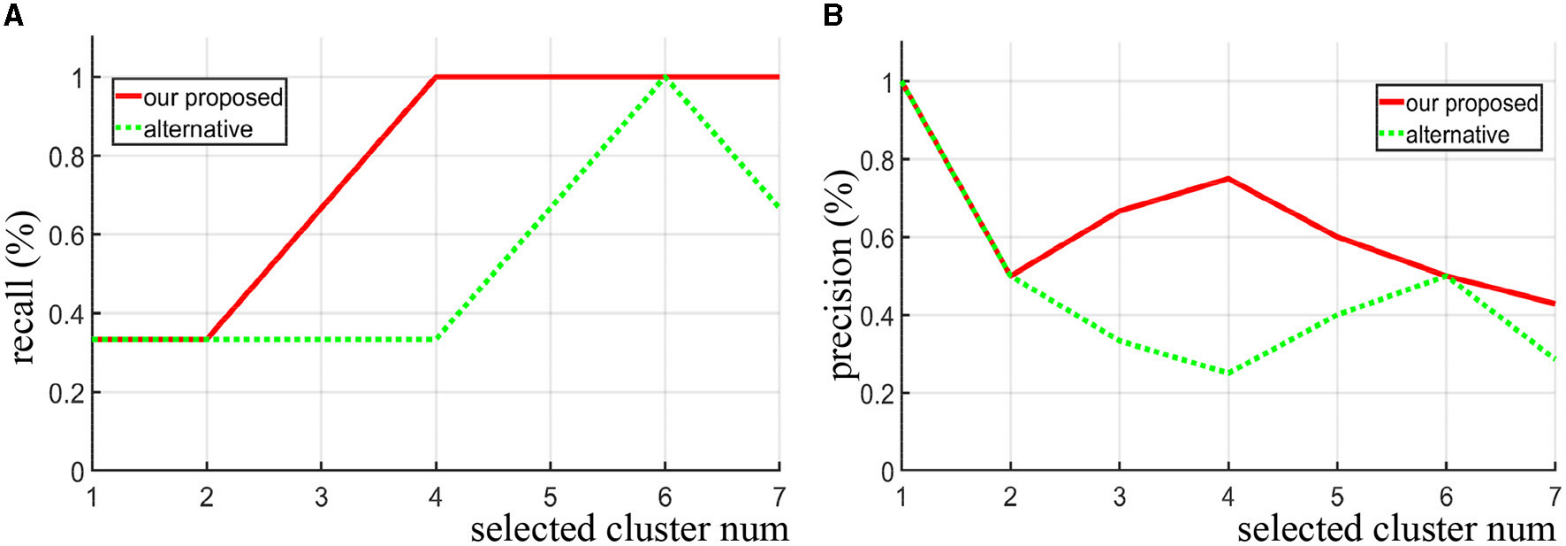
Given different selected cluster number *N*_*sel*_, the recall curve **(A)** and precision curve **(B)** of our algorithm and the alternative method are compared on the synthetic data.

For the synthetic data, we directly use the precision and recall curve since we know where the true discriminative features are. Precision (also called positive predictive value) is the fraction of discriminative clusters among the retrieved clusters, while recall (also known as sensitivity) is the fraction of discriminative clusters that have been retrieved over the total discriminative clusters. As shown in Figures 3A, B, when the same number of clusters is selected, our proposed method achieves both higher recall and precision score compare to the alternative approach (area under the two curves). Notice that when four clusters are selected (*N*_*sel*_ = 4), all the three true discriminative clusters can be detected. When increasing the number of selected clusters, our proposed method still maintained a high recall rate, while the alternative method does not seem to improve. Even when the number of clusters set to seven, the recall rate drops instead. In contrast to the alternative approach, our method is more likely to detect the real discriminative regions as increasing the number of selected clusters.

### 3.3. Real fMRI data I—Haxby dataset

Based on the simulation experiments, we use a well-established public dataset, Haxby, a study of face and object representation in human ventral temporal cortex (Haxby et al., 2001). The work innovatively incorporates the idea of structured sparsity into the framework of stability selection (randomized structure sparsity, RSS in short). The author compared their results with a range of classical univariate voxel selection methods and multi-voxel pattern identification methods, which showed relatively fewer false positives and confirmed the validity (higher predictive accuracy) of selected voxels. These methods include *T*-test, *l*2-SVM, *l*2 Logistic Regression, *l*1-SVM, *l*1 Logistic Regression, randomized *l*1 logistic regression, Smooth Lasso (Hebiri and Van de Geer, 2011) and TV-L1 (Gramfort et al., 2013) and Randomized Ward Logistic (Gramfort et al., 2012).

The Haxby dataset consists of six subjects with 12 runs per subject (dataset can be downloaded at http://data.pymvpa.org/datasets/haxby2001/). In each run, the subjects passively viewed grayscale images of eight object categories, grouped in 24*s* blocks separated by rest periods. Each image was shown for 500 *ms* and was followed by a 1,500 *ms* inter-stimulus interval. Full-brain fMRI data were recorded with a volume repetition time of 2.5 *s*. Then a stimulus block was covered by roughly nine volumes. For a complete description of the experimental design, fMRI acquisition parameters, and previously obtained results, check the reference on their website (Haxby et al., 2001; Hanson et al., 2004). In this paper, we use the fMRI data of subjects one to five and classifying the “House” and “Cat”, which is a classic case for animal vs. non-animal classification. Preprocessing of the data consisted of motion correction using SPM 12, normalization and registration to the Montreal Neurological Institute (MNI) to facilitate inter-subject segmentation, removal of linear trends in each session, etc. There is no smoothing operation on the data. In the process of coregistration, the structural data is coregistered with functional data. Due to the missing of structural data, subject six is excluded from the analysis.

To have a fair comparison, we use the same parameter settings for RSS and our method: In particular, the number of clusters *N*_*C*_ = 200, the connection radius σ_*d*_ = 3, the block size 3 × 3 × 3, the times of spatial randomization iterations *N*_*K*_ = 200, subsampling fraction α_*col*_ = 0.01, fixed regularization parameter λ = 0.3. Several additional parameter is used in our approach for cross verification *N*_*CV*_ = 20, α_*row*_ = 0.9 and sampling quality control α_*K*_ = 0.3, *N*_*sel*_ = 15 is chosen for this study. This study was not interested in the activities of the cerebellum and vermis regions, therefore these regions were masked to rule out for consideration.

**Figure 4:**
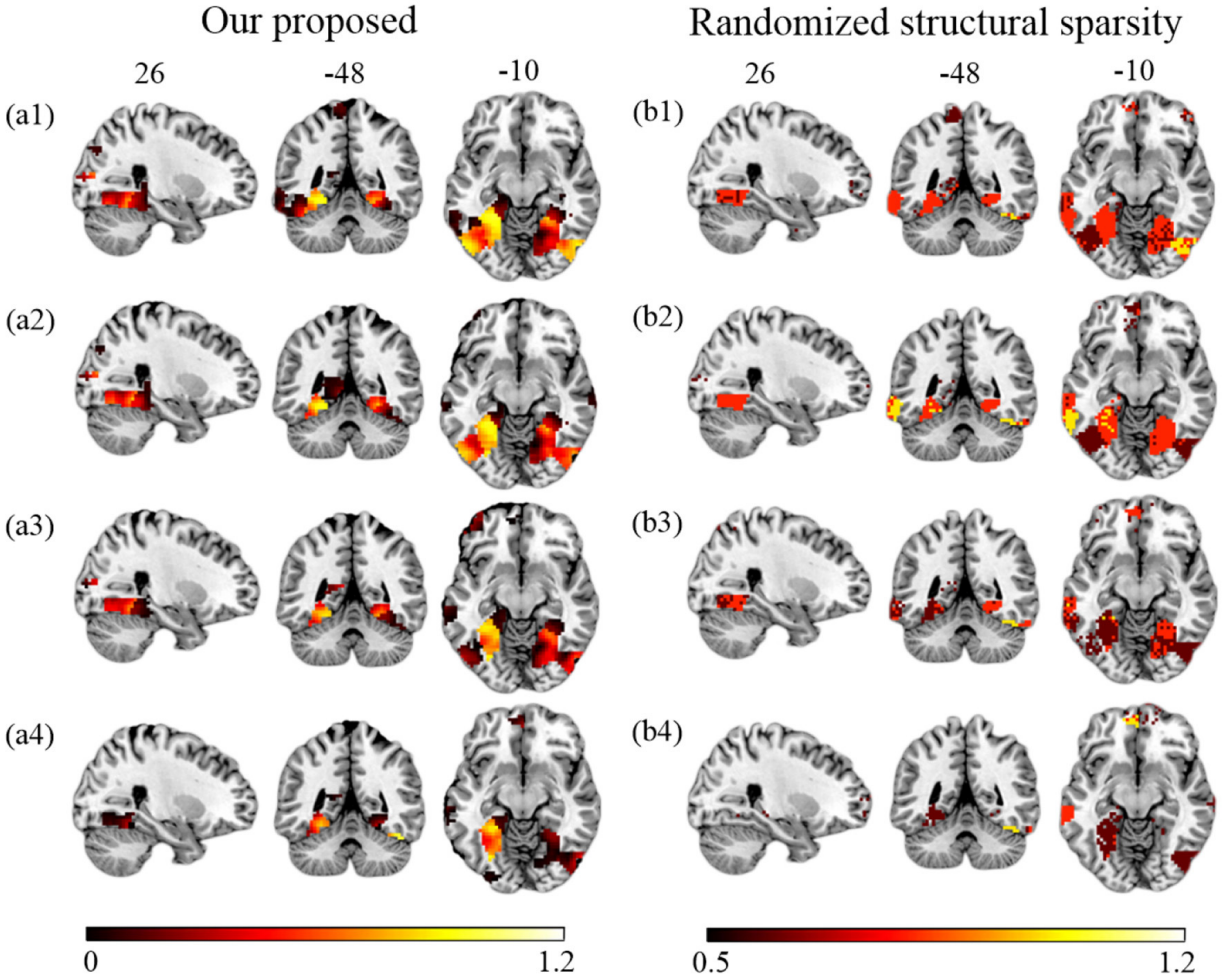
Brain maps for discriminative voxels as estimated on Haxby data (Cat vs. House). **(Left)** EVR maps (unthresholded) by our proposed approach. **(Right)** Maps of binominal test result for RSS, thresholded at 0.5. Both approaches used exactly the same amount of data for comparison (1) six sessions (the first 1/2) of five subjects; (2) four sessions (the first 1/3) of five subjects; (3) three sessions (the first 1/4) of five subjects; (4) two sessions (the first 1/6) of five subjects.

First, we compare the performance of our proposed method and RSS when decreasing the number of training samples. We use the first T sessions for training, which correspond to 1/2, 1/3, 1/4, and 1/6 of the data (*T* = 6, 4, 3, 2) for each subject. In Figure 4, we show the EVR maps from our method (a1–a4, not thresholded), and binominal test results of score maps across subjects (b1–b4, thresholded at 0.5). It shows that our proposed algorithm locates stable discriminative voxels at bilateral fusiform and inferior temporo-occipital even with fewer training samples (see the pattern in a3 and a4).

**Figure 5:**
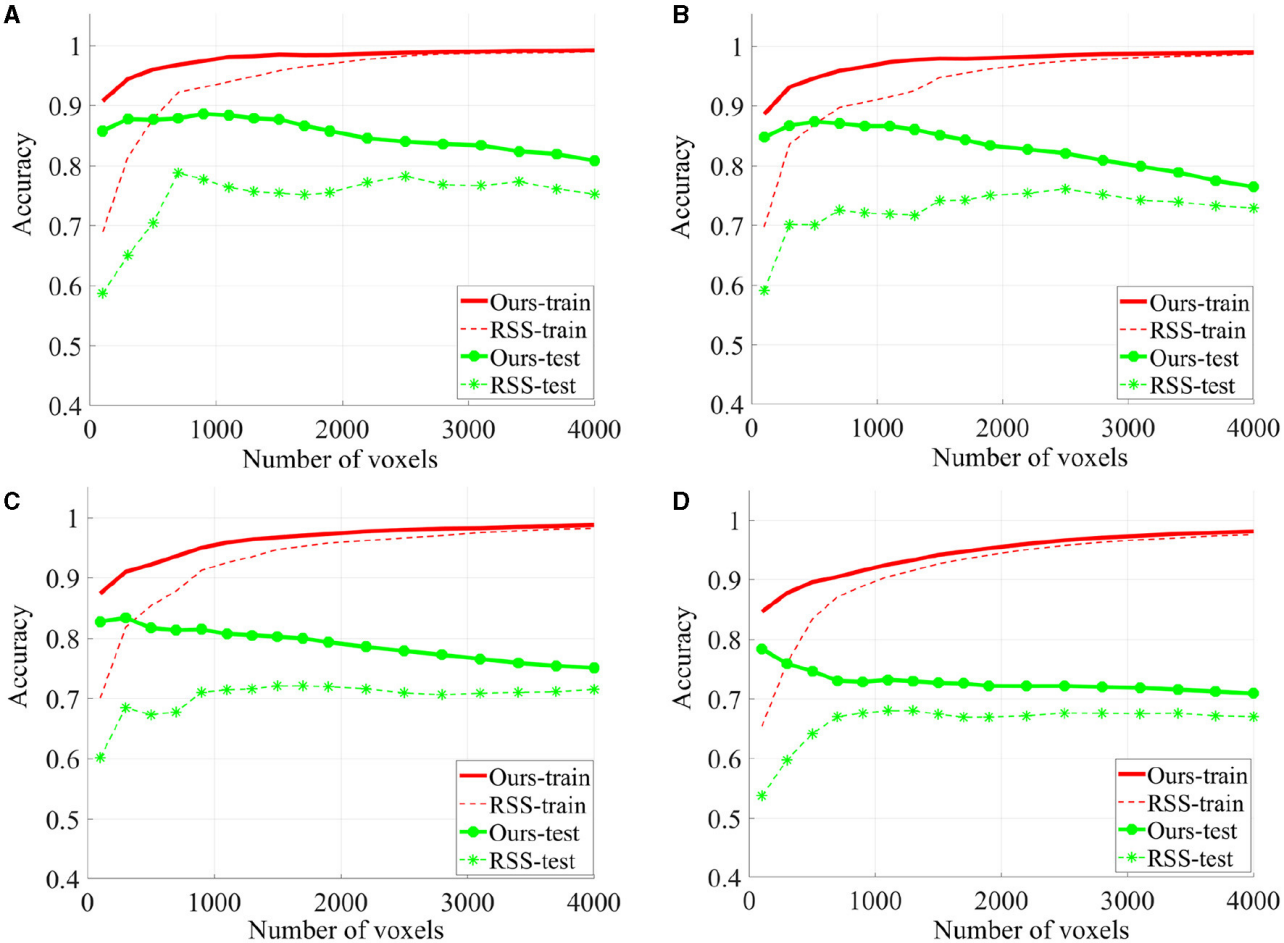
The classification accuracy based on 4-fold cross verification on House & Cat each curve is estimated on each individual and then averaged across folds and subjects. Six sessions **(A)**, four sessions **(B)**, three sessions **(C)**, and two sessions **(D)** are used for training.

To evaluate the quality of the identified discriminative voxels, we conducted 4-fold cross validation using a linear l2-SVM classifier for both our proposed method and RSS. Figure 5 illustrates the changes in training and testing accuracy as the number of voxels increases. The reported curves are averaged across subjects and four times cross verification. Our method allowed for early identification of discriminative voxels. However, as more voxels were included (since the exact number of discriminative voxels is unknown), there was an increase in irrelevant voxels and noise. This led to a decline in the accuracy curve. On the other hand, the alternative method did not effectively identify discriminative voxels. With an increasing number of voxels, both irrelevant and truly relevant voxels were included, resulting in a flat curve. It is important to note that our method consistently outperforms the comparison method, as our curve consistently remains higher than the RSS curve.

### 3.4. Real fMRI data II—Fear conditioned dataset

After conducting experiments on synthetic data and commonly used public datasets, we initially tested and validated the robustness and sensitivity of the parameters of the proposed method. In general, our proposed approach outperforms the alternative approach in terms of its strength in recovering the discriminative pattern reliably when reducing the number of training samples, as well as keeping the sensitivity of individual specificity. Further, we exploratively conduct experiments on an earlier fMRI small sample dataset and then visualize the results. The data were recorded from a differential aversive conditioning study in which Gabors of one orientation were occasionally paired with an electric shock (see Petro et al., 2017; Ji et al., 2019, for details). For the habituation block, participants were instructed that they would not feel any shock but to fixate on the patterns. During the acquisition block, participants were informed that they would intermittently feel a cutaneous electric shock during the experiment but were not instructed as to the contingencies of the shock administration. The extinction phase was also uninstructed, such that participants were not told that no more shocks were to be given. The data reported here include 40 total trials per phase per participant. Each trial consisted of one of the two gratings being presented for 5, 100*ms*, during which its phase was alternated every 100 *ms*. An inter-trial interval (ITI) consisted of an initial gray cross (37.5 *cd*/*m*^2^; 1° of visual angle) presented in the middle of the screen for a random duration between 0 − 8 *s* followed by a white cross (149.0 *cd*/*m*^2^) for a duration of 3 *s*, immediately preceding trial onset with Gabor patch presentation.

The Data were acquired during gradient-echo echo-planar imaging sequence with a 3T Philips Achieva scanner [echo time (TE), 30 *ms*; repetition Time (TR), 1.98 *s*; flip angle, 80°; slice number, 36; field of view, 224 mm; voxel size, 3.5 × 3.5 × 3.5 *mm*^3^; matrix size 64 × 64]. Preprocessing of BOLD fMRI data was completed using SPM12. We followed the standard preprocessing routines: slice timing correction, head movements realigning, normalization and resampled to a spatial resolution of 3 × 3 × 3 *mm*^3^. Images were smoothed using a Gaussian kernel with a full-width at half-maximum of 6 *mm*. Low-frequency temporal drifts were removed from the BOLD data using a 1/128 *Hz* high-pass filter.

Following our previous work (Petro et al., 2017), the general linear models (GLMs) were constructed to extract features. The GLM aimed to model the ssVEP-BOLD coupling over the entire experiment. Thus, all trials were modeled separately using a GLM, which consisted of a sequence of boxcar functions in which the start was synchronized with the onset of each stimulus and width equal to the duration of each trial. Each boxcar function was then convolved with a canonical hemodynamic response function. Six additional regressors describing participants' head movements, as determined during preprocessing, were added to this design matrix to account for head movements during the scanning process. Excluding the motion components from the coefficient matrix, the single-trial coefficients are next used as features for decoding.

**Figure 6:**
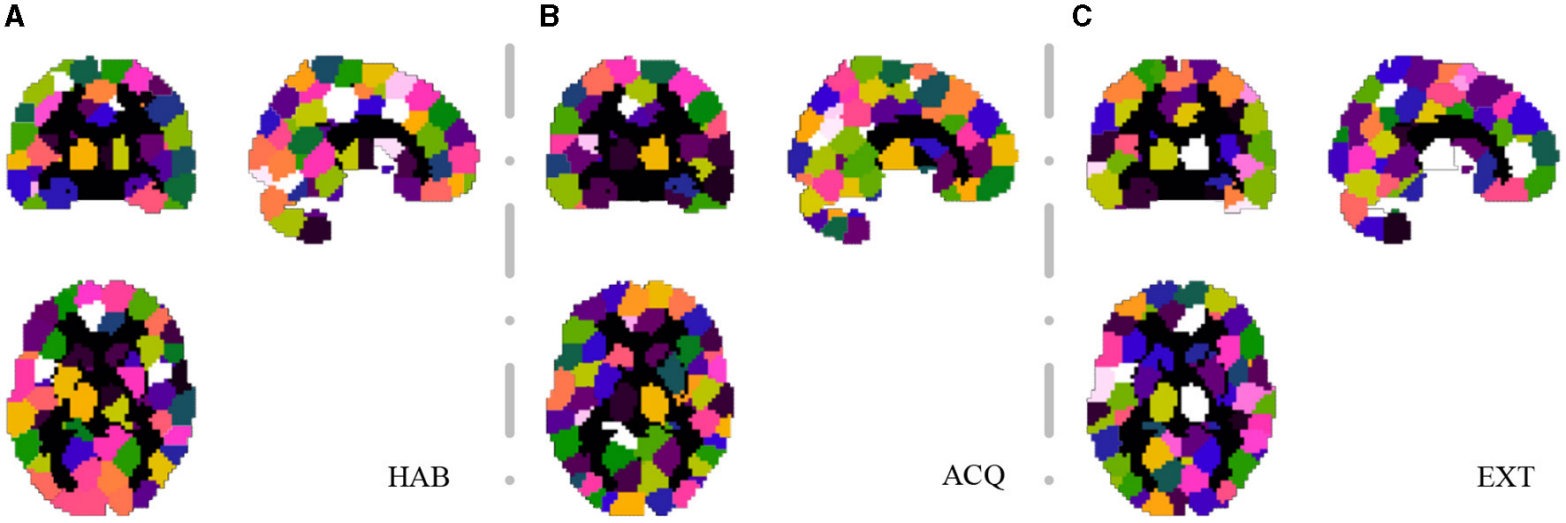
Segmentation snapshot of three experimental sessions: **(A)** habituation (HAB), **(B)** acquisition (ACQ), **(C)** extinction (EXT). Different areas are marked with different colors, for a total of 200 brain partitions.

For the SHV scheme, the number of selected clusters is crucial, and as the number of of *N*_*sel*_ increases, the random overlap of clusters also increases. If *N*_*sel*_ is too large, it will reduce the sensitivity of the cluster voting rate and EVR. However, if *N*_*sel*_ is too small, it will result in more false negatives. We recommend selecting this parameter based on prior knowledge. In this study, we choose *N*_*sel*_ = 40 based on the previous analysis of EEG-ssVEP (Ji et al., 2018, 2019). Segmentation was performed based on the homogeneity of functional time series and feature correlations, as described in Section 2.1. Since this study did not interested in the activities of the cerebellum and vermis regions, these regions were masked out (AAL template 91-116). For the current data set, we select 200 for *N*_*C*_ and set the connection radius σ_*d*_ as 3 voxels. The results are reported in Figure 6. Although prediction accuracy may not be the sole criteria for selecting a model, it generally indicates that some of these voxels are truly discriminative when the prediction accuracy is high. To evaluate the quality of the discovered discriminative voxels, we employed a linear l2-SVM classifier (Hebiri and Van de Geer, 2011). Although not required, for all three experimental sessions, we pre-saved random seeds for block subsampling and cross-validation to ensure the same settings were made for all subjects to facilitate comparison. We set the times of spatial randomization iterations *N*_*K*_ = 1, 000, times of cross verification *N*_*CV*_ = 20, subsampling fraction α_*col*_ = 0.015 and α_*row*_ = 0.9, sampling quality control ratio α_*K*_ = 0.3.

**Figure 7:**
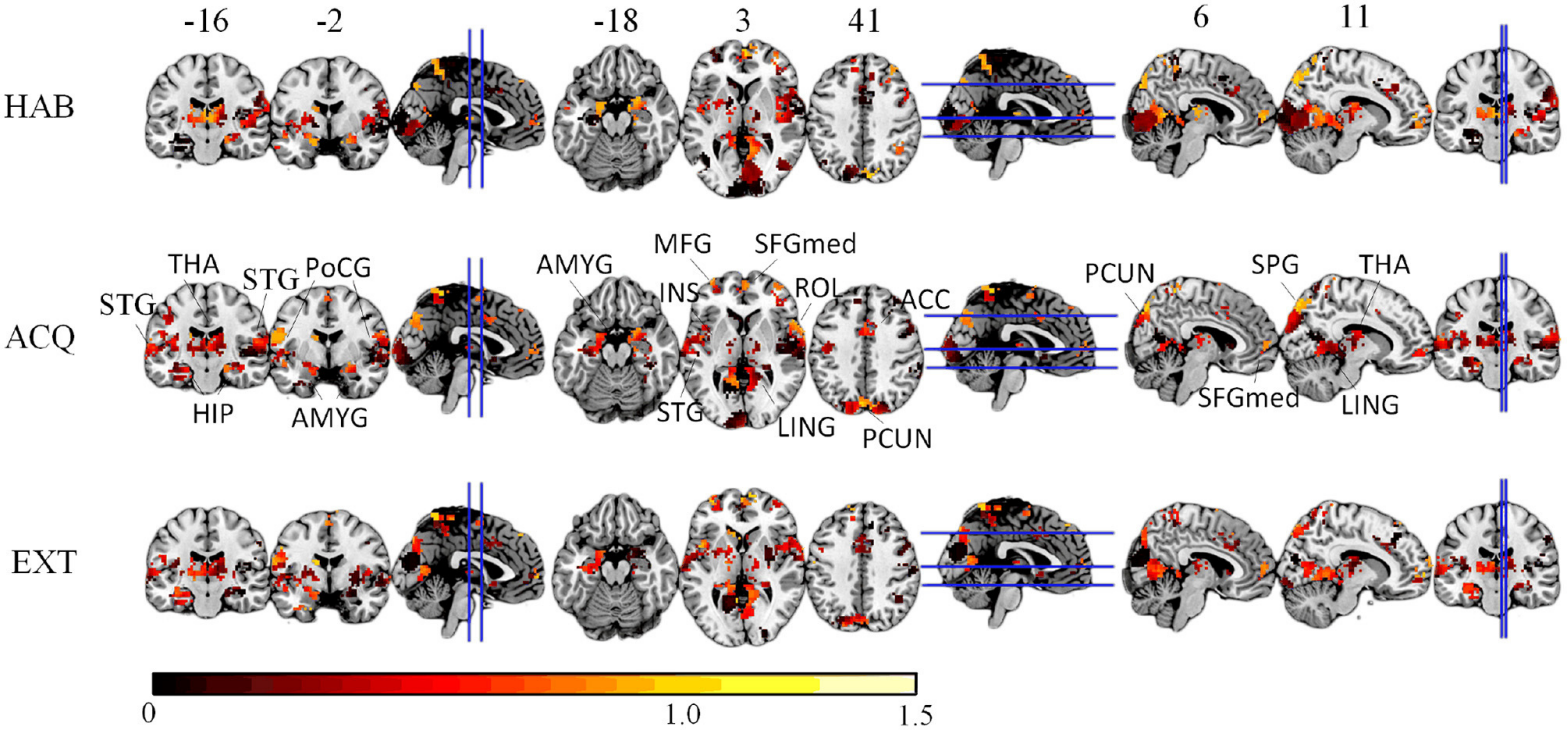
The EVR brain maps (unthreholded), which is the computed by averaging EVR across subjects.

**Figure 8:**
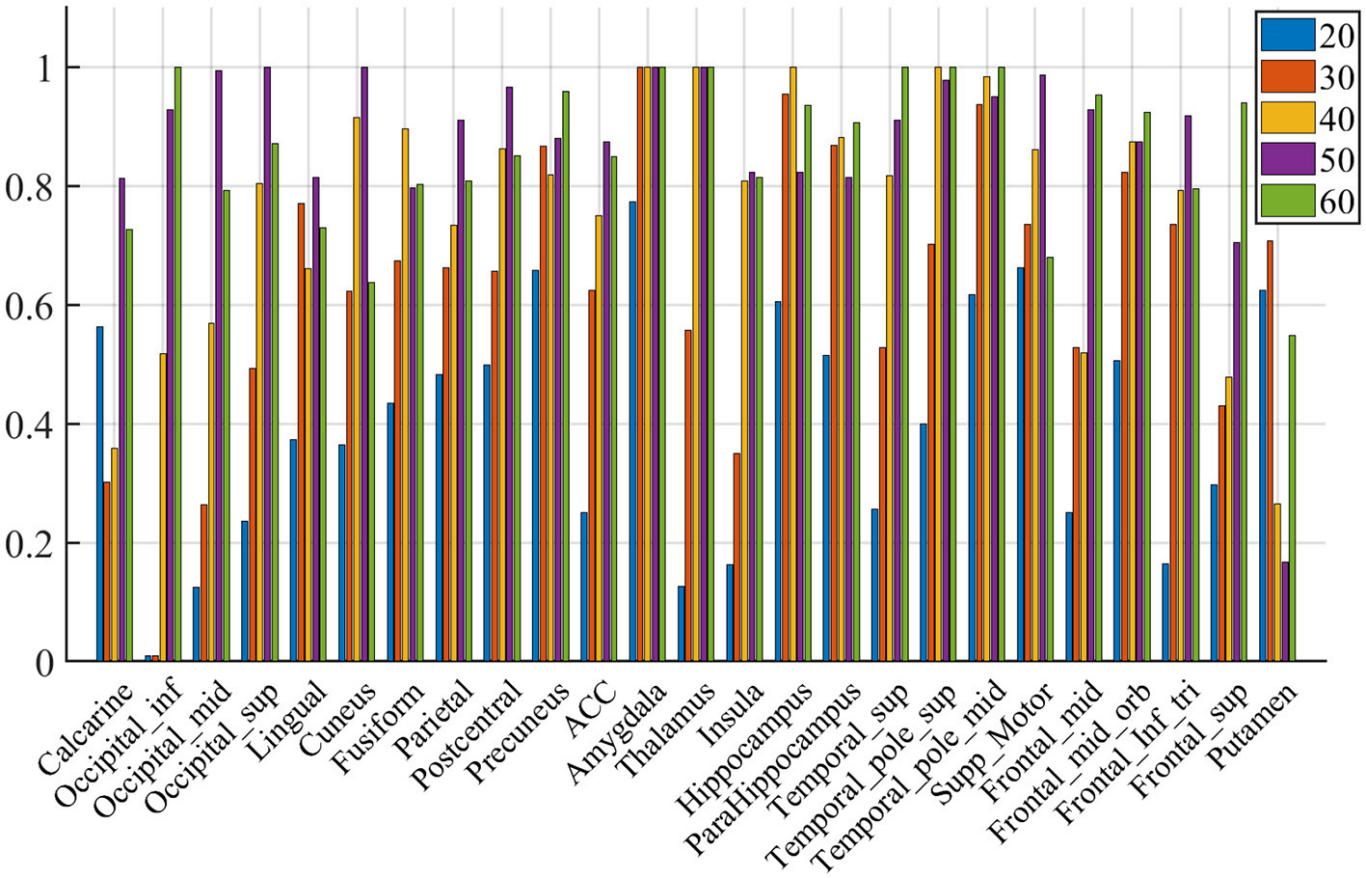
Voting rates changes with different *N*_*sel*_, in the proportion of the vote across nine subjects. The results are for acquisition for demonstration purposes only.

We compute the EVR using Equation (6), the brain maps are shown in Figure 7 which are not thresholded for visualization purpose. Table 1 shows detail information for acquisition session, including the corresponding coverage—the ratio between the number of non-zero EVR voxels and the total number of voxels in that region—to indicate the region size of discriminative features, the “Peak-EVR" and “MNI" show the peak location and peak intensity of each listed region. From the EVR map, the discriminative voxels across three experimental sessions largely pointed to the same regions, including the visual cortical areas such as calcarine, lingual, cuneus, occipital, and fusiform gyrus, and a set of functionally connected brain regions such as the superior frontal gyrus (orbital and medial part), postcentral, the superior temporal gyrus, the superior and middle temporal pole, precuneus and parietal gyrus, anterior cingulate cortex, insula, amygadala and thalamus. For acquisition, ROIs got the highest regional coverage are: the calcarine, lingual, superior temporal gyrus, hippocampus and parahippocampus, thalamus, as well as middle frontal gyrus, parietal, precuneus, postcentral and fusiform gyrus for their absolute number of discriminative voxels. To test the influence of *N*_*sel*_ to the results of cluster voting rates, Figure 8 is added. For most regions, increasing the number of selected clusters yield larger overlap across subjects.

**Table 1:** The region size/coverage of discriminative features, the peak EVR value and the corresponding MNI coordinates are listed for each ROI during the acquisition session.

**Location**	**Region size (coverage)**	**MNI**	**Peak-EVR**
Calcarine	478/1,285	−6, −49, 5	0.96
Inferior occipital	7/548	−15, −100, −7	0.19
Middle occipital	113/1,592	−30, −85, 35	0.39
Superior occipital	278/840	24, −76, 47	0.98
Lingual	425/1,266	−6, −52, 2	0.90
Cuneus	204/817	6, −82, 41	1.00
Fusiform	207/1,415	−18, −43, −10	0.85
Parietal	375/2,344	9, −82, 50	1.00
Postcentral	243/2,261	−54, −4, 20	0.98
Precuneus	282/2,029	−6, −76, 41	1.00
ACC	29/390	0, 8, 41	0.71
Amygdala	57/136	24, −1, −10	0.92
Thalamus	316/663	−15, −10, 17	0.94
Insula	127/1,101	−45, 8, −7	0.84
Hippocampus	192/562	24, −16, −13	0.86
ParaHippocampus	154/634	21, 5, −25	0.92
Superior temporal	507/1,640	−51, −10, −4	0.90
Superior temporal pole	67/764	63, 14, −1	0.96
Middle temporal pole	86/2,782	−51, −61, 17	1.00
Supplementary motor	45/1,367	−6, 5, 80	0.77
Middle frontal	325/2,947	48, 50, 5	0.95
Middle frontal, orbital	58/538	21, 65, −10	0.88
Inferior frontal, triangular	50/1,435	51, 44, 5	0.83
Superior frontal	104/2,266	−36, 62, 2	0.86
Putamen	25/597	−30, −19, 8	0.28

**Figure 9:**
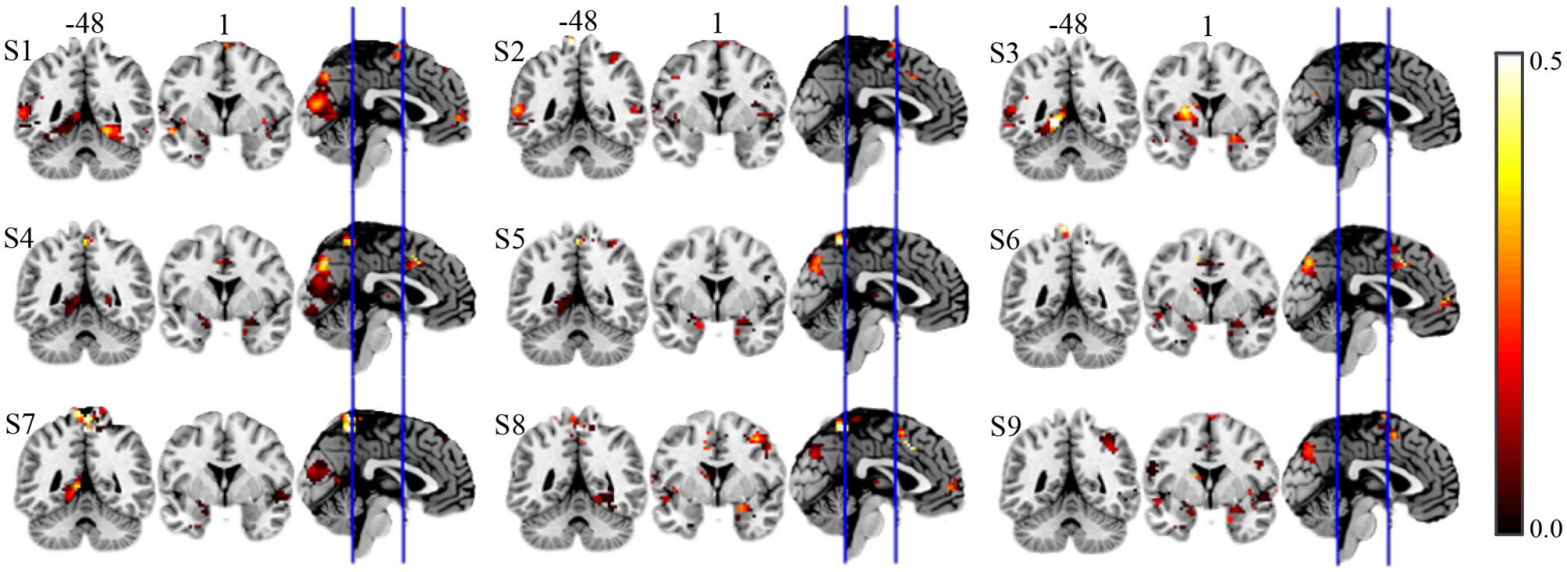
EVR results of nine subjects of alternative approach on real fMRI data.

To quantify the relative importance of discriminative voxels, we compute the mean effective vote ratio (EVR, see Eqution 6) across nine subjects. The resulted brain maps are shown in Figure 7, which are not thresholded for visualization purposes, meaning that the zeros displayed are actually zeros. By visual inspection, it is easy to detect the significant discriminative area. For the convenience of comparison, we also illustrate the EVR results of nine subjects in Figure 9, that only data from a single subject are used.

Lastly, we compute the stability index $\bar{O}$ and the averaged test accuracy $\bar{R}$ both for our proposed method and alternative method. The results are compared for 3 experimental sessions: habituation (HAB), acquisition (ACQ), and extinction (EXT), as shown in Table 2. Compared to the alternative approach, the voxels selected by our method achieves higher test correct ratio/prediction accuracy. As indicated by the stability index, our results yield solutions that more consistent and concentrated between individuals. Meanwhile, the test accuracy stably increases across experimental sessions and suggests heightened discrimination between threat and safety in visual regions in acquisition compared to habituation.

**Table 2:** The stability index and the averaged test accuracy of our proposed method and alternative method across three experimental sessions, habituation (HAB), acquisition (ACQ), and extinction (EXT), respectively.

**Session**	** ${\bar{O}}_{alter.}$ **	** ${\bar{O}}_{our}$ **	** ${\bar{R}}_{alter.}$ **	** ${\bar{R}}_{our}$ **
HAB	0.12	0.86	0.62	0.65
ACQ	0.20	0.87	0.65	0.69
EXT	0.22	0.87	0.70	0.73

## 4. Discussion

We conduct numerical experiments on synthetic data and commonly used public dataset to test and cross-validate our proposed method. The results show that explicitly accounting for stability/groupwise consistency during the model optimization can mitigate some of the instability inherent in sparse methods. In particular, using the mixed *l*1 and *l*2 norm as a joint optimization criterion allows pooling data from multiple subjects and can lead to solutions that are concentrated in a few brain regions between different individuals. The number of selected candidate features is allowed to be much larger when incorporating group structure, which allows us a more global search among brain regions. Introducing groupwise regularization as an additional optimization criterion may offer promise for future methodological developments in the analysis of small-sample fMRI dataset.

These results are in line with recent predictive coding models (Rao and Ballard, 1999; Friston, 2005; Spratling, 2008), in which separate populations of neurons within a cortical region code the current estimate of sensory causes (predictions) and the mismatch between this estimate and incoming sensory signals (prediction error). Here, we did not manipulate the prior expectation of the occurrence or omission of stimuli (grating stimuli were present in all trials), but the likelihood of the stimulus having a certain feature (i.e., orientation) and it's followed by an electric shock. Thus, expectancy about the events during CS− (safe outcome) vs. CS+ (shock will occur after a fixed time interval) is learned as the experimental session progresses.

Finally the proposed method also resulted in findings that converge with other approaches, and with theoretical and computational models or fear conditioning and object recognition. Specifically, we found heightened discrimination between threat and safety in visual regions in acquisition compared to habituation, and we found increasing sparsification as fear learning progressed. It is worthy to note that, the prediction accuracy (the correct ratio on test set) may be significantly above chance, but far from perfect. This indicates that the code contains some linearly decodable information, but claims of linear separability may be difficult to evaluate as it would require attributing the substantial proportion of errors to limitations of the measurements (noise and subsampling), rather than to a lack of linear separability of the neuronal activity patterns. In the case of object perception, the method proposed in this thesis resulted in more robust and spatially coherent regions, illustrating its potential usefulness and applicability to a wide range of questions in cognitive neuroscience.

## Data availability statement

The original contributions presented in the study are included in the article/supplementary material, further inquiries can be directed to the corresponding author.

## Ethics statement

The studies involving humans were approved by Behavioral/NonMedical Institutional Review Board, University of Florida. The studies were conducted in accordance with the local legislation and institutional requirements. The participants provided their written informed consent to participate in this study.

## Author contributions

NZ and HJ: conceptualization. HJ and XZ: methodology. HJ and AK: investigation. HJ, XZ, and AK: writing. BC, ZY, and AK: supervision. HJ: funding acquisition. All authors had full access to all the data in the study and take responsibility for the integrity of the data and the accuracy of the data analysis. All authors contributed to the article and approved the submitted version.
